# Pathogenesis of obstructive sleep apnea in individuals with the COPD + OSA Overlap syndrome versus OSA alone

**DOI:** 10.14814/phy2.14371

**Published:** 2020-02-15

**Authors:** Jeremy E. Orr, Christopher N. Schmickl, Bradley A. Edwards, Pamela N. DeYoung, Rebbecca Brena, Xiaoying S. Sun, Sonia Jain, Atul Malhotra, Robert L. Owens

**Affiliations:** ^1^ Division of Pulmonary, Critical Care, and Sleep Medicine University of California San Diego La Jolla CA USA; ^2^ Sleep and Circadian Medicine Laboratory Department of Physiology Monash University Melbourne VIC Australia; ^3^ Turner Institute for Brain and Mental Health Monash University Melbourne VIC Australia; ^4^ Division of Biostatistics and Bioinformatics Department of Family Medicine and Public Health University of California San Diego La Jolla CA USA

**Keywords:** COPD, lung, OSA

## Abstract

Overlap syndrome (OVS) is the concurrence of chronic obstructive pulmonary disease (COPD) and obstructive sleep apnea (OSA), and is associated with poor outcomes. We hypothesized that physiological changes in COPD may affect the pathogenesis of OSA in important ways. We therefore sought to measure the anatomical and nonanatomical OSA traits in individuals with OVS and compare to those with OSA alone. Patients with established OVS were recruited, along with age, gender, and BMI matched OSA only controls. Smoking and relevant comorbidities or medications were excluded. Subjects underwent baseline polysomnography followed by an overnight physiological research study to measure the OSA traits (V_eupnea_, V_arousal_, V_passive_, V_active_, and loop gain). Fifteen subjects with OVS and 15 matched controls with OSA alone were studied (overall 66 ± 8 years, 20% women, BMI 31 ± 4 kg/m^2^, apnea‐hypopnea index 49 ± 36/hr). Mixed‐modeling was used to incorporate each measurement (range 52–270 measures/trait), and account for age, gender, and BMI. There were no significant differences in the traits between OVS and OSA subjects, although OVS subjects potentially tolerated a lower ventilation before arousal (i.e., harder to wake; *p* = .06). Worsened lung function was significantly associated with worsened upper airway response and more unstable breathing (*p* < .05 for all). Consistent differences in key OSA traits were not observed between OVS and OSA alone. However, worse lung function does appear to exert an influence on several OSA traits. These findings indicate that a diagnosis of OVS should not generally influence the approach to OSA, but that lung function might be considered if utilizing OSA trait‐specific treatment.

## Graphical abstract

This is the first study to comprehensively measure both anatomical and non‐anatomical OSA traits during sleep in those with COPD + OSA (Overlap syndrome) and compare them to those with OSA alone. No consistent differences were found between the two groups, while worsened lung function did impact upper airway function and control of breathing. These findings have implications towards mechanisms underlying OSA, as well as management of sleep apnea.

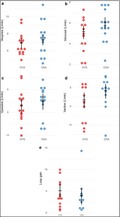

## 1 INTRODUCTION

The co‐occurrence of chronic obstructive pulmonary disease (COPD) and obstructive sleep apnea (OSA) has been termed the overlap syndrome (OVS) (Flenley, 1985; Owens & Malhotra, 2010). OVS has been associated with poor quality of life (Mermigkis et al., 2007) and cardiovascular consequences (Sajkov & McEvoy, 2009; Sharma et al., 2013; Taranto‐Montemurro et al., 2016). Patients with OVS have higher mortality than those with COPD alone (Marin, Soriano, Carrizo, Boldova, & Celli, 2010), and the adverse effects of COPD and OSA may be synergistic rather than additive (Kendzerska et al., 2019). While epidemiological data have not supported a link between mild COPD and OSA (Bednarek, Plywaczewski, Jonczak, & Zielinski, 2005; Sanders et al., 2003), in cohorts of higher COPD severity the prevalence of OSA appear high relative to the general population (Lopez‐Acevedo, Torres‐Palacios, Elena Ocasio‐Tascon, Campos‐Santiago, & Rodriguez‐Cintron, 2009; Soler et al., 2015), although these studies have lacked a control group and the validity of OSA diagnostic criteria in lung disease is not known. Thus questions arise regarding mechanisms underlying OSA in COPD and whether they differ from OSA without COPD.

It is increasingly appreciated that OSA is influenced by several traits, including anatomical factors (i.e., upper airway collapsibility), as well as nonanatomical factors (upper airway muscle responses, respiratory‐related arousability from sleep, and control of breathing) (Owens et al., 2015; Schmickl, Owens, Edwards, & Malhotra, 2018). These traits might be influenced by COPD via several mechanisms, leading to differences in the pathophysiology of OVS compared to those with OSA alone.

With respect to anatomical factors, stability of the upper airway is dependent on traction from the trachea, thus increases in lung volumes can improve upper airway collapsibility (i.e., stiffen the airway) (Owens, Malhotra, Eckert, White, & Jordan, 2010; Van de Graaff, 1988). Consequently, increased lung volumes in COPD may have a protective effect on upper airway closing pressure and thus may reduce the apnea–hypopnea index (Biselli et al., 2015; Krachman et al., 2016). However, a loss of elastic recoil related to emphysema may decrease tracheal traction forces, and thus the net effect of COPD on upper airway collapsibility is difficult to predict. Moreover, large differences in upper airway collapsibility between OVS and OSA alone would not be expected since a sufficiently collapsible upper airway is requisite for OSA, suggesting that the nonanatomical OSA traits may be of greater interest.

The potential influence of COPD on the nonanatomical traits is suggested by several observations. Patients with COPD often have symptoms of sleep disturbance and objectively poor sleep (Cormick, Olson, Hensley, & Saunders, 1986; Kinsman et al., 1983), particularly in the presence of air trapping and hyperinflation (Krachman et al., 2005; Kwon, Wolfe, Lu, & Kalhan, 2009). This tendency toward frequent arousals might translate to a tendency to wake up in response to inspiratory flow limitation from upper airway collapse; that is, a low respiratory arousal threshold. Systemic myopathy seen in COPD and/or local effects from inhaled corticosteroids or smoking might translate to impairments in upper airway dilator muscle function (Agusti et al., 2002; Meurice, Marc, & Sériès, 1995; Teodorescu et al., 2009). Lastly, COPD appears to increase neural respiratory drive (Jolley et al., 2009), which might lead to instability in breathing control. On the other hand, mechanical limitations and a mechanically disadvantaged diaphragm might have the opposite effect by limiting overshoots in ventilation (Scano et al., 1995).

Importantly, differences in pathogenesis between OVS and OSA alone could have important implications for management. Therapy with continuous positive airway pressure (CPAP) has been associated with improved outcomes; however, many patients are unable to use CPAP long‐term. Alternative upper airway‐targeted therapies such as oral appliances, surgery, and hypoglossal nerve stimulation have not been studied in OVS patients and in clinical practice are generally not offered to such patients. There is a growing interest in the use of sedative‐hypnotics and other pharmacotherapy for OSA; data regarding the utility versus risk of such an approach in OVS patients is conflicting (Donovan et al., 2019; Holmedahl, Overland, Fondenes, Ellingsen, & Hardie, 2015). Another consideration is the use of oxygen which is frequently provided to hypoxemic COPD patients during wakefulness and sleep; oxygen may be helpful in the context of unstable ventilatory control but could potentially prolong respiratory events in some patients (Alford, Fletcher, & Nickeson, 1986). These considerations highlight the importance of understanding the underlying pathogenesis in order to better assess whether such alternative OSA treatments may be useful or even safe for patients with OVS.

On the basis of this conceptual framework, we performed a comprehensive assessment of both the anatomical and nonanatomical traits in individuals with OVS and those with OSA alone, with the hypothesis that the OSA traits differ between these two groups.

## 2 METHODS

The study was approved by the University of California San Diego Human Research Protection Program (IRB#161873). All subjects signed informed consent prior to participating in any research.

### 2.1 Subjects

Men and women with previously established OSA were prospectively recruited from a University pulmonology and sleep clinic, prior research studies, and the local population via advertisements. Inclusion criteria included patients in the age group of 45–75 years with a prior diagnosis of OSA and a apnea–hypopnea index > 5/hr. Patients with a prior clinical diagnosis of concurrent COPD (i.e., individuals with OVS) were specifically recruited. Asthma‐COPD overlap was not specifically excluded. We aimed to case‐match each OVS subject with an OSA‐alone subject on the basis of gender, age ± 5 years, and BMI ± 3 kg/m^2^. Exclusion criteria were use of medications known to affect control of breathing (i.e., narcotics and sedatives), daytime supplemental oxygen use, recent hospitalization or respiratory infection (<3 months), body mass index (BMI) >36 kg/m^2^, active major medical problems (symptomatic coronary artery disease, congestive heart failure, cirrhosis, end‐stage renal disease, psychiatric disease), active tobacco use, or >3 oz. per night alcohol use.

### 2.2 Baseline testing

Following enrollment, we obtained a complete medical history and questionnaires (Epworth Sleepiness Scale, Pittsburg Sleep Quality Index, and SF‐36). Spirometry, lung volumes by plethysmography, and diffusing capacity were performed according to ATS standards (Miller et al., 2005). Spirometry was completed during one of the study nights.

Baseline polysomnography was obtained on all subjects. Signals included electroencephalography, electro‐oculography, tibial electromyography, electrocardiography, thermistor, nasal pressure, thoracic and abdominal effort signals, and fingertip pulse oximetry (Nonin LifeSense), as well as transcutaneous capnography (Sentec SDM V‐Sign). All signals were sampled at 125 Hz and were acquired using a 1,401 digital‐analog converter and Spike2 acquisition software (Cambridge Electronic Design Ltd). Subjects went to sleep at their usual bedtime and were asked to sleep in the supine position for at least 6 hr.

### 2.3 Physiological testing

**Figure 1:**
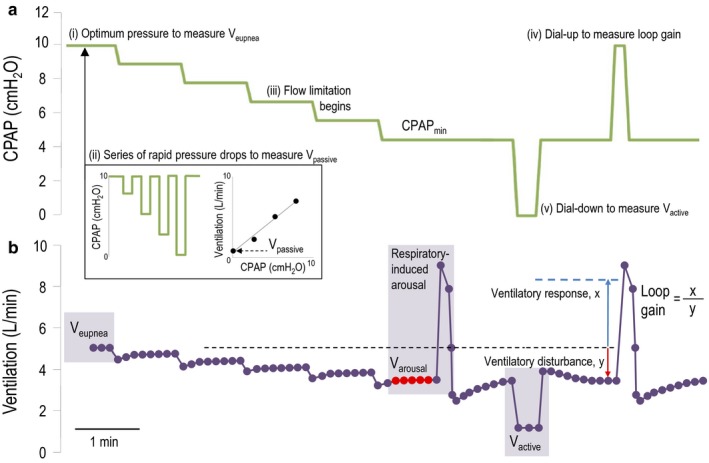
Measurement of OSA traits via a Series of Pressure Changes. OSA traits are measured by manipulating CPAP (Panel a) during supine non‐rapid eye movement (NREM) sleep and measuring the resultant changes in ventilation (Panel b). (i) Minute ventilation is taken over 30–60 s while on optimal CPAP settings (i.e., holding pressure) to measure V_eupnea_; (ii) The pressure is rapidly dropped to sequentially lower pressures. Minute ventilation (for V_passive_) and peak inspiratory flow (for P_crit_) is taken from the third through fifth breaths following a drop. Regression is used to determine ventilation at atmospheric pressure for V_passive_, and CPAP level at onset of zero peak inspiratory flow for P_crit_. (iii) CPAP is then gradually lowered until flow limitation starts and arousals occur intermittently. Ventilation just prior to arousal is defined as V_arousal_. During stable breathing between arousals under this maximally‐increased respiratory drive, CPAP is dialed down or up from this level to obtain V_active_ (v) and loop gain (vi), respectively. Minute ventilation taken is from the second and third breath following a rapid drop from the minimum tolerable CPAP level and regression is used to determine ventilation at atmospheric pressure for V_active_. Loop gain is the ventilatory response (first breath overshoot in ventilation above V_eupnea_) divided by the ventilatory disturbance (preceding five breath reduction in ventilation below V_eupnea_). Adapted from (Edwards et al., 2016)

On a separate night within 4 weeks of the baseline polysomnography (with clinical stability), subjects underwent a physiological sleep study using a previously validated technique, as described in prior publications, and summarized in Figure 1 (Edwards et al., 2016; Owens et al., 2015; Wellman et al., 2013). Briefly, subjects were instrumented for polysomnography as above, without nasal sensors. Subjects were fitted with a nonvented CPAP mask with end‐tidal capnography (Vacumed) and mask pressure monitoring, which was attached to a heated pneumotachometer (Validyne), exhalation port, and standard CPAP tubing. A modified CPAP machine (ResMed) capable of providing rapidly changing pressures ranging from +20 to −20 cm H_2_O was connected. The patient was asked to sleep in the supine position. Once asleep, the subjects were titrated to a therapeutic CPAP level (i.e., holding pressure) to abolish flow limitation. First, brief sequential drops to subholding pressure for five breaths were performed to measure passive airway characteristics. Second, a slow, stepwise dial down in small decrements was performed until the minimum tolerable CPAP pressure causing intermittent arousals was obtained. The pressure was then dropped to lower pressure levels for three breaths to determine the maximally stimulated upper airway characteristics. Finally, the pressure was dialed up to slightly above therapeutic pressure for three breaths to quantify the ventilatory response to a ventilatory disturbance (i.e., loop gain).

### 2.4 Analysis

Polysomnography was scored by a Registered Polysomnographic Technologist (RPSGT) according to American Academy of Sleep Medicine standards (hypopnea definition: clear decrease in airflow lasting >10 s followed by 3% desaturation and/or arousal) (Sleep‐Related Breathing Disorders in Adults, 1999).

Data analysis of the physiology study was performed in MATLAB R2018a (The Mathworks), in order to quantify breath‐by‐breath ventilation (“removing” varying amounts of unintentional leak via signal processing), and incorporation of associated breath‐by‐breath data such as CPAP level. The physiological traits which we directly measured and calculated are:

V_eupnea_: Ventilation during sleep in the absence of upper airway obstruction (i.e., on therapeutic CPAP pressure), such that ventilation should match baseline ventilatory drive.
V_passive_ and P_crit_: Both are measures of collapsibility of the upper airway, determined at baseline ventilatory drive (i.e., without accumulation of any additional drive that might result from prolonged upper airway obstruction). V_passive_ measures ventilation at atmospheric pressure (zero cm H_2_O) under such nonactivated airway conditions, while P_crit_ measures the pressure at which inspiratory flow goes to zero under nonactivated airway conditions.
V_arousal_ and Ventilatory arousal threshold (ArTh): Both are measures of the tendency to wake from sleep due to inspiratory flow limitation. V_arousal_ measures the minimum ventilation that can be sustained without arousal during steady‐state conditions. Ventilatory drive accumulates under such conditions, leading to increases in intrathoracic pressure swings; a high V_arousal_ indicates a tendency to wake up with minimal increase in intrathoracic pressure swings. The ArTh is a calculated value, using the LG and V_eupnea_ data to estimate the ventilatory drive that is present at the ventilation measured by V_arousal_.
V_active_ and Upper airway gain (UAG): Both are measures of the ability of the upper airway to dilate in response to increases in respiratory drive. V_active_ measures ventilation at atmospheric pressure (zero cm H_2_O) under maximally tolerated increased respiratory drive (i.e., at the same point in drive as V_arousal_ and ArTh). The UAG is a calculated value, using V_passive_, V_active_, V_eupnea_, and LG to determine the increase (or decrease) in ventilation achieved using an increase in respiratory drive.
Loop Gain (LG): Measures instability in ventilatory control, by measuring the ratio of ventilatory response to a steady‐state ventilatory disturbance. The disturbance is the increase in drive that results from steady‐state inspiratory flow limitation conditions, and response is determined by suddenly alleviating the flow limitation.

### 2.5 Statistical analysis

Baseline characteristics were compared between OVS and OSA groups. Continuous variables were examined using an independent sample *t*‐test or Mann–Whitney *U* test, as appropriate by visual inspection of the sample distribution, while dichotomous variables were examined using Fisher's exact test.

The difference between each trait in the OVS group and OSA alone group was compared using linear mixed‐modeling, incorporating every measurement for the trait of interest, and considering each subject as a random effect parameter. In order to decrease the variance, all models were adjusted a priori for fixed effects of age, gender, and BMI. Parameter estimates are reported in the text as mean ± *SE*.

Finally, linear mixed modeling (adjusting for age, gender, and BMI) was also used to examine the influence of lung function measures on the traits, including forced expiratory volume in 1 s (FEV1), forced vital capacity (FVC), residual volume (RV), functional residual capacity (FRC), total lung capacity (TLC), and the ratio between RV and TLC (RV/TLC). Lung function variables were analyzed as continuous (i.e., original) values, but reported as per 10% change to improve interpretability.

A *p* value of <.05 was considered significant. The traits that were directly measured (V_eupnea_, V_arousal_, V_passive_, V_active_, and loop gain) were considered primary outcomes; computed measures (arousal threshold and upper airway gain) were considered secondary given the inherent variability when combining multiple variables together. Statistical analysis was performed in R (version 3.5.2; http://www.r-project.org) and SPSS Statistics 25 (IBM). Figures were generated using the ggplot2 package in R.

## 3 RESULTS

### 3.1 Subjects

Two COPD subjects were excluded due to absence of OSA on baseline sleep study despite reported history of OSA. About 15 OVS subjects and 15 well‐matched OSA controls were included. Baseline demographics and pulmonary function testing measures are shown in Table 1. Three subjects in the OVS group with self‐reported COPD were found to have an FEV1/FVC ratio of >0.7. For baseline polysomnography, five subjects with OVS were using nocturnal oxygen at home with their CPAP machines, of which three did not use supplemental oxygen during the baseline PSG. One subject was started on oxygen midway through the night; oximetry data reported are while off oxygen. One OVS subject desaturated below 85% immediately after falling asleep; oximetry data were excluded from analysis. All physiology study nights were performed without supplemental oxygen. Baseline polysomnography results are reported in Table 2.

**Table 1:** Demographic, polysomnographic, and lung function characteristics of the study population

	OVS (*n* = 15)	OSA alone (*n* = 15)	*p* value
Age (years)	67 ± 6	65 ± 7	.44
Male gender, *n* (%)	12 (80)	12 (80)	>.99
BMI (kg/m^2^)	30.0 ± 3.6	31.1 ± 3.7	.41
ESS	7 ± 5	7 ± 5	.91
Pack‐years smoking	32 [52]	0 [20]	**.02**
Awake seated SpO2 (%)	94 ± 1	94 ± 3	.93
Inhaled corticosteroids (%)	7 (47%)	0 (0%)	**<.01**
FEV1 (%predicted)	60 ± 24	92 ± 17	**<.01**
GOLD I: *n* (%)	3 (20)	N/A	N/A
GOLD II: *n* (%)	7 (47)		
GOLD III: *n* (%)	4 (27)		
GOLD IV: *n* (%)	1 (7)		
FVC (% predicted)	79 ± 15	89 ± 16	.10
FEV1/FVC ratio	56 ± 16	78 ± 5	**<.01**
MMEF 25%–75%	39 ± 36	109 ± 36	**<.01**
RV (% predicted)	140 ± 44	104 ± 29	**.02**
FRC (% predicted)	126 ± 36	101 ± 16	**.02**
TLC (% predicted)	102 ± 16	94 ± 13	.12
RV/TLC ratio (%)	49 ± 11	39 ± 7	**<.01**
DLCO (% predicted)	65 ± 28	81 ± 15	.07

Note

Data are shown as mean ± *SD* or median [IQR]. *p* values <.05 are shown in bold.

Abbreviations: BMI, body mass index; ESS, Epworth sleepiness scale; FEV1, forced expiratory volume in 1 s; FRC, functional residual capacity; FVC, forced vital capacity; GOLD, Global Initiative for Chronic Obstructive Lung Disease; MMEF 25%–75%, maximal mid‐expiratory flow; RV, residual volume; RV/TLC, ratio of RV to TLV; TLC, total lung capacity.

**Table 2:** Baseline polysomnography data

	OVS (*n* = 15)	OSA alone (*n* = 15)	*p* value
Sleep efficiency (%)	62 ± 18	75 ± 14	**.04**
%NREM	87 ± 8	88 ± 10	.86
%REM	13 ± 8	12 ± 10	.86
AHI (events/hr)	41 ± 29	57 ± 32	.17
NREM AHI (events/hr)	41 ± 30	57 ± 33	.17
REM AHI (events/hr)	33 ± 21	54 ± 41	.11
OAI (events/hr)	9 [14]	14 [31]	.63
CAI (events/hr)	1 [1]	0 [7]	.95
Mean SpO2 (%), wake	92 ± 1	93 ± 2	.11
Mean SpO2 (%), sleep	90 ± 2	92 ± 3	.07
Mean SpO2 (%), NREM	91 ± 2	92 ± 3	.11
Mean SpO2 (%), REM	87 ± 5	92 ± 4	**.03**
TST SpO2 < 90% (%)	28 [52]	7 [33]	.11
Nadir desaturation (%)	78 ± 7	81 ± 5	.22
Mean TcCO2 (mmHg), sleep	41 ± 5	40 ± 12	.82

Note

Data are shown as mean ± *SD* or median [IQR]. *p* values <.05 are shown in bold.

Abbreviations: AHI, apnea–hypopnea index; CAI, central apnea index; NREM, non‐rapid eye movement; OAI, obstructive apnea index; REM, rapid eye movement; SpO2, Pulse oximetry oxyhemoglobin saturation; TcCO2, transcutaneous carbon dioxide; TST, total sleep time.

**Figure 2:**
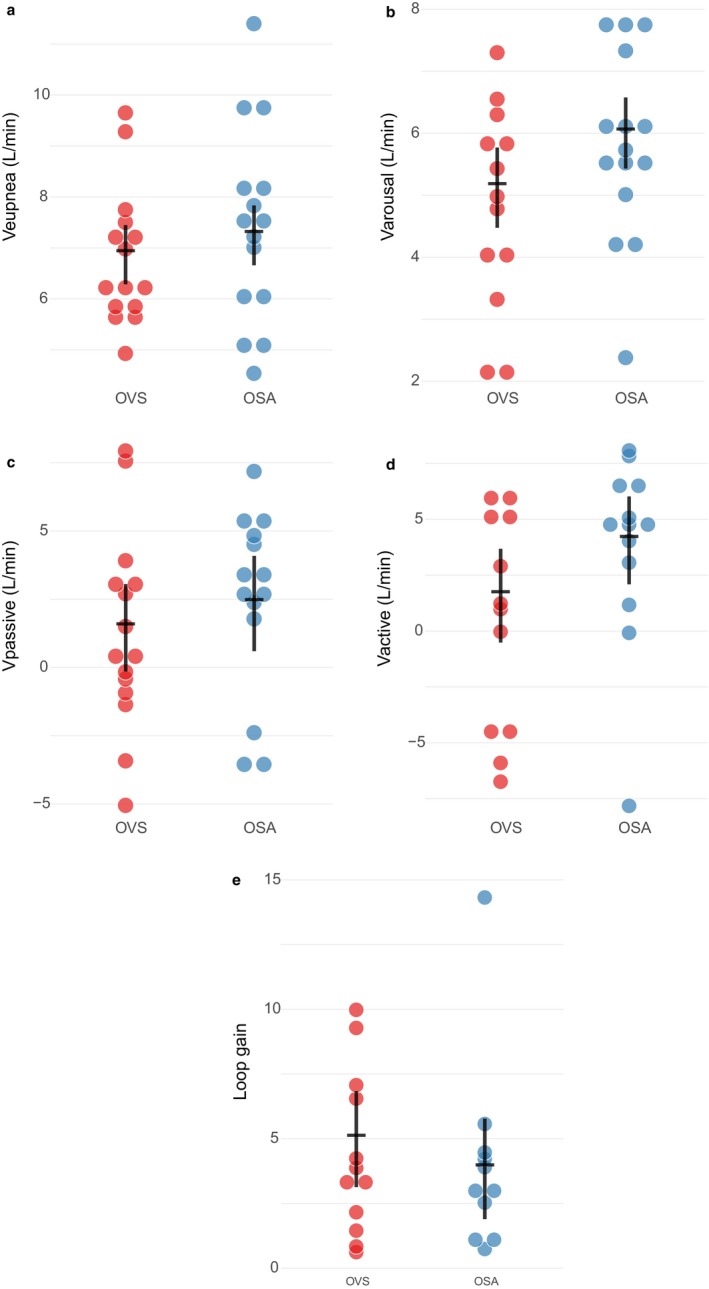
Comparison of OSA traits for subjects with OVS versus OSA alone. Circles represent mean values of each OSA trait for each subject. The dot and whisker plot shows the mixed modeling group estimated mean with associated [95% confidence interval]. There was no significant difference in (a) V_eupnea_ (6.9 [6.3–7.4] vs. 7.2 [6.7–7.8] L/min; *p* = .38), (b) V_arousal_ (5.1 [4.5–5.8] vs. 6.0 [5.4–6.6] L/min; *p* = .06), (c) V_passive_ (1.5 [0–3.1] vs. 2.3 [0.6–4.1] L/min; *p* = .48), (d) V_active_ (1.6 [−0.5 to 3.7] vs. 4.1 [2.1–6.0] L/min; *p* = .11), or (e) Loop gain (5.0 [3.1–6.8] vs. 3.8 [1.9–5.8]; *p* = .43)

*V_eupnea_:* A total of 270 measurements across 30 subjects were obtained (127 in 15 OVS, and 143 in 15 OSA alone). In the adjusted model, there was no significant difference in eupneic ventilation between OVS and OSA subjects in V_eupnea_ (Table 3). Individual mean values and group estimates for V_eupnea_ are shown in Figure 2. There was no association between V_eupnea_ and any lung function measures (Table 4).

**Table 3:** Measured OSA traits in subjects with OVS versus OSA alone

	OVS	OSA alone	Difference	*p* value
	Mean ± *SEM*	Mean ± *SEM*	Mean [95% CI]	
V_eupnea_ (L/min)	6.9 ± 0.3	7.2 ± 0.3	−0.4 [−1.2, 0.5]	.38
V_arousal_ (L/min)	5.1 ± 0.3	6.0 ± 0.3	−0.9 [−1.7, 0.0]	**.06**
V_passive_ (L/min)	1.5 ± 0.8	2.3 ± 0.9	−0.9 [−3.3, 1.5]	.48
P_crit_ (cm H_2_O)	−1.7 ± 0.7	−2.7 ± 0.7	1.0 [−1.0, 3.0]	.33
V_active_ (L/min)	1.6 ± 1.1	4.1 ± 1.0	−2.5 [−5.4, 0.4]	.11
Loop gain (dimensionless)	5.0 ± 0.9	3.8 ± 1.0	1.1 [−1.7, 4.0]	.43
Arousal threshold (L/min)	17.6 ± 2.3	11.9 ± 2.4	5.7 [−1.0, 12.4]	.12
Upper airway gain (dimensionless)	0.1 ± 0.3	0.8 ± 0.3	−0.7 [−1.5, 0.2]	.14

Note

Models are adjusted for age, gender, and BMI. Coefficients with *p* < .10 are bolded.

**Table 4:** Relationship between lung function parameters and OSA traits in adjusted mixed model analysis

	FEV1	FVC	RV	FRC	TLC	RV/TLC
V_eupnea_	*β* = .05	*β* = −.03	*β* = .06	*β* = .03	*β* = .15	*β* = .19
	[−0.11, 0.22]	[−0.31, 0.25]	[−0.05, 0.18]	[−0.14, 0.21]	[−0.18, 0.48]	[−0.24, 0.63]
	*p* = .54	*p* = .83	*p* = .28	*p* = .72	*p* = .39	*p* = .40
V_arousal_	*β* = .10	*β* = .0	*β* = −.01	*β* = −.03	*β* = −.11	*β* = −.04
	[−0.08, 0.27]	[−0.29, 0.31]	[−0.13, 0.11]	[−0.23, 0.16]	[−0.47, 0.26]	[−0.52, 0.43]
	*p* = .30	*p* = .95	*p* = .87	*p* = .73	*p* = .58	*p* = .86
V_passive_	*β* = −.03	*β* = −.23	*β* = −.20	*β* = .04	**β = −.94**	*β* = −.37
	[−0.51, 0.44]	[−1.03, 0.58]	[−0.52, 0.12]	[−0.46, 0.54]	**[−1.84, −0.04]**	[−1.67, 0.93]
	*p* = .89	*p* = .59	*p* = .24	*p* = .87	***p* = .05**	*p* = .58
V_active_	*β* = .34	*β* = .19	***β *= −.55**	*β* = −.42	***β* = −1.58**	***β* = −1.86**
	[−0.24, 0.93]	[−1.23, 0.73]	**[−0.90,−0.19]**	[−1.01, 0.17]	**[−2.57, −0.58]**	**[−3.39, −0.33]**
	*p* = .26	*p* = .73	***p* < .01**	*p* = .18	***p* < .01**	***p* = .03**
Loop gain	***β* = −.62**	***β* = −.81**	***β* = .43**	*β* = .28	*β* = .37	***β* = 2.05**
	**[−1.06, −0.17]**	**[−1.65, 0.03]**	**[0.05, 0.81]**	[−0.32, 0.87]	[−0.79, 1.51]	**[0.53, 3.57]**
	***p* = .01**	***p* = .07**	***p* = .04**	*p* = .37	*p* = .55	***p* = .02**

Abbreviations: FEV1, forced expiratory volume in 1 s; FVC, forced vital capacity; RV, residual volume; FRC, functional residual capacity; TLC, total lung capacity, RV/TLC, ratio of RV to TLV. Data are shown as nonstandardized regression coefficient per 10% increase in percent predicted and associated [95% CI], except RV/TLC which is reported as per 10% absolute change [95% CI]. Models are adjusted for age, gender, and BMI, and include data from all subjects (OVS and OSA alone). Coefficients with *p* < .10 are bolded.

*V_arousal_:* A total of 249 measurements across 28 subjects were obtained (98 in 13 OVS, and 151 in 15 OSA‐alone). In the adjusted model, there was no significant difference in ventilation just prior to arousal in patients with OVS compared to OSA alone (Table 3, Figure 2). There was no association between V_arousal_ and any lung function measures (Table 4).

*V_passive_ and P_crit_:* A total of 52 acceptable values were obtained in 29 subjects (31 in 15 OVS, and 21 in 14 OSA alone). There was no significant difference in V_passive_ between OVS and OSA (Table 3, Figure 2). There was no statistically significant association between V_passive_ and any lung function measures (Table 4).

P_crit_ was also not significantly different between OVS and OSA (Table 3). There was no significant association between P_crit_ and any lung function measures (*p* > .10; data not shown).

*V_active_:* A total of 148 V_active_ measurements were obtained in 25 subjects. (75 in 12 OVS, and 73 in 13 OSA alone). There was no significant difference in V_active_ between OVS and OSA (Table 3, Figure 2). A lower V_active_ was significantly associated with increased residual volume, increased total lung capacity, and increased RV/TLC ratio (Table 4). There was no association with other lung function measures.

*Loop gain:* A total of 52 LG measurements across 23 subjects were obtained (26 in 12 OVS, and 26 in 11 OSA alone). There was no significant difference in LG between OVS and OSA subjects (Table 3, Figure 2). A higher LG was significantly associated with a lower FEV1, higher RV and RV/TLC ratio, and a trend with lower FVC (Table 4).

*Arousal Threshold:* A total of 205 arousal threshold measurements across 22 subjects were calculated (94 in 12 OVS, and 111 in 10 OSA‐alone) from individual V_arousal_ measurements and the subject's mean loop gain. The arousal threshold was not significantly different in those with OVS compared to OSA (Table 3). There was no association between arousal threshold and any lung function measures (*p* > .10; data not shown).

*Upper airway gain:* A total of 88 upper airway gain values across 20 subjects were calculated (50 in 10 OVS, and 38 in 10 OSA alone). There was no significant difference in UAG between those with OVS compared to OSA (Table 3). There was no significant association between UAG and any lung function measures (*p* > .10; data not shown).

## 4 DISCUSSION

We did not find consistent differences in important anatomic and nonanatomic traits influencing OSA pathogenesis between individuals with OVS and those with OSA alone. We studied a group of OVS that reflects a typical clinical population with generally moderate COPD, and thus we conclude that in general, obstructive sleep apnea in OVS does not appear fundamentally different disease than “run‐of‐the‐mill” OSA.

However, we did observe a strong relationship between several important OSA traits and lung function parameters, which is a novel finding. Specifically, we found: 1) reduced upper airway response (V_active_) in those with indicators of air trapping (higher RV, TLC, and RV/TLC ratio), and 2) increased loop gain in those with airflow obstruction (lower FEV1, higher RV and RV/TLC ratio).

The finding of decreased upper airway response in relation to worsening air trapping is consistent with one prior study that indicated decreased upper airway dilation in response to CO_2_ amongst those with COPD compared to controls (Meurice et al., 1995). Potential explanations include an issue with mechanical linkage between lower and upper airways, or confounding factors related to COPD severity that also might impact the upper airway. Pharyngeal dilator neuromyopathy has been implicated amongst some groups with OSA, including those with obesity (Sands et al., 2014). Potential risk factors for similar issues in those with COPD includes prior smoking, systemic myopathy, and local effects of inhaled corticosteroids (Teodorescu et al., 2009). Indeed, many of our OVS subjects were using inhaled corticosteroids—particularly those with worsened COPD—and thus additional study in this area appears warranted.

The finding that loop gain increases with worsening airflow obstruction is contrary to the hypothesis that limitations to airflow in such patients would effectively dampen any large swings in ventilation. On the other hand, worsening COPD severity is associated with increased hypoxemia. During events, hypoxemia in combination with hypercapnia is a particularly potent respiratory stimulus. In addition, chronic hypoxemia (both intermittent and sustained) has been shown to increase respiratory control sensitivity via neuroplasticity (Dempsey & Smith, 2014). Although this elevated loop gain might propagate respiratory events in OSA, it may also help to prevent prolonged events (particularly in the presence of a highly collapsible upper airway), and thus may be at least partially adaptive. This concept is consistent with a recent study finding where there was increased ventilatory drive amongst those with OVS compared to both COPD and OSA alone (He et al., 2017).

As expected, we did not see major differences in passive upper airway collapsibility between OVS and OSA. We suspect this is due to the fact that a collapsible upper airway is requisite for OSA to be present. Similar to a previous study (Biselli et al., 2015), we did not find a difference in P_crit_ between OVS and OSA alone, but in contrast we did not see a relationship between P_crit_ and FRC, perhaps because we considered FRC relative to body size (i.e., percent predicted). We did observe an association between increased total lung capacity and lower V_passive_, which did not meet significance but encompassed a potentially important effect size. Based on the physiological differences between P_crit_ (i.e., closing pressure, relying on peak inspiratory flow) and V_passive_ (measuring ventilation at atmospheric pressure) (Landry et al., 2017), a similar P_crit_ but lower V_passive_ might be due to inspiratory negative effort dependence (Owens et al., 2014) or a different site of upper airway collapse (Azarbarzin et al., 2017).

We found a suggestion of decreased respiratory‐related arousability amongst those with OVS compared to OSA alone, not accounted for by changes in lung function. While the data did not meet a statistical cutoff, it clearly did not support our initial hypothesis that patients with COPD would be more easily awoken. A tolerance to lower levels of ventilation prior to arousal would promote longer events and may result in an overnight loading of CO_2_ (Berger et al., 2000). Most of our patients did not have substantial hypercapnia, but a lower V_arousal_ might provide an explanation for the finding that patients with OVS are predisposed to hypercapnia beyond what can be explained by their lung function (Resta et al., 2002). Additional study of arousability in OVS patients with hypercapnia is thus warranted given that these patients appear to be at particularly high risk for adverse outcomes (Jaoude, Kufel, & El‐Solh, 2014; Kuklisova, Tkacova, Joppa, Wouters, & Sastry, 2017).

To our knowledge, this is the first prospective study to measure comprehensively OSA pathogenesis in OVS subjects during sleep and compare to OSA alone. The strengths of this study include a prospective design with detailed physiological measurements taken during sleep using a well‐validated technique for determining OSA traits. We used a matched design and analysis adjusting for variables known to affect OSA pathogenesis—age, gender, and BMI. In addition, we used a mixed‐modeling approach, rather than a comparison by subject means, in order to capitalize on all the measurements as well as to take into account factors such as within‐individual variability and variable measurements per each subject. Thus we believe the estimates are a good reflection of the differences in OSA pathogenesis attributed to the presence of COPD and changes in lung function.

The number of subjects was relatively modest, which may have limited the power of this study to detect differences in OSA traits. However, each subject had multiple measurements of each trait, and we used a mixed‐modeling approach to improve the robustness of our findings. This was an exploratory study without prior data from which to perform a sample size calculation, and we refrained from additional enrollment after reaching our target in attempt to reach statistical significance. Additional subjects may have changed the results, although our observed confidence intervals provide the most probable range of the true difference between the OVS and OSA alone populations. Prior literature provides some clinical context to determine if this range includes clinically important differences. Edwards et al. found that responders (i.e., 50% reduction in AHI to a level <10/hr) to an oral appliance had a V_passive_ that was approximately 2.5 L/min higher than those who did not respond (Edwards et al., 2016). In our data, the 95% CI for the difference in V_passive_ between OVS and OSA alone was mostly less than this value <2.5 L/min, suggesting that even with our sample size we are unlikely to miss differences in V_passive_ between OVS and OSA that would be clinically important. Ongoing studies by our laboratory and others will provide additional data regarding clinically significant differences in OSA traits.

There were several other limitations to this study. The degree of obstruction in those with OVS was moderate, which might limit generalizability to more severe COPD, despite our analysis of the impact of variable levels of lung function. We also used standard seated pulmonary function testing to evaluate COPD physiology that might account for changes in OSA traits. However, lung volumes and airflow are likely to change while supine, which might limit the precision of our conclusions. In addition, we only included individuals with a prior diagnosis of OSA. Since screening tools for OSA in those with COPD are not well established, those diagnosed with OVS might differ from those with OSA, which could lead to occult confounders, despite our matching for factors known to affect OSA pathogenesis. Finally, by studying only individuals with OSA, we cannot make general conclusions about these traits in non‐OSA COPD subjects, that is, we cannot definitively say whether COPD predisposes or protects from OSA. We matched and accounted for factors known to impact OSA pathogenesis (age, gender, and BMI), which strengthens the null hypothesis that the OVS and OSA groups would have the same traits if COPD does not affect OSA pathogenesis. Nonetheless, in order to have OSA, one must have compatible traits; for example, if COPD leads to reduced upper airway collapse, those “protected” from OSA by nature of their COPD would have been “missed” by our study.

In conclusion, we did not find consistent differences in the pathogenesis of OSA amongst those with concurrent COPD (i.e., overlap syndrome, OVS) compared to OSA alone. Arousal responses were not elevated in OVS as we had suspected, but rather might be lower in some individuals. We observed an association between worsening lung function and worsened upper airway response, as well as more unstable control of breathing, although we cannot determine causality in this cross‐sectional study. These findings indicate that in general, the clinical approach to OVS should not differ from OSA alone. However, treatments aimed at specific OSA traits may be influenced by poor lung function and the presence of COPD in some individuals. This study emphasizes the need for mechanistic research into factors that influence OSA pathogenesis at an individual level.

## CONFLICT OF INTEREST

The authors have no relevant conflict of interest to disclose.
